# Supplementary figures and images for: Dual RNA-Seq Profiling Unveils Mycoparasitic Activities of Trichoderma atroviride against Haploid Armillaria ostoyae in Antagonistic Interaction Assays
Source: Microbiol Spectr. 2023 May 4;11(3):e04626-22. doi: 10.1128/spectrum.04626-22 (PMC10269595; doi:10.1128/spectrum.04626-22)

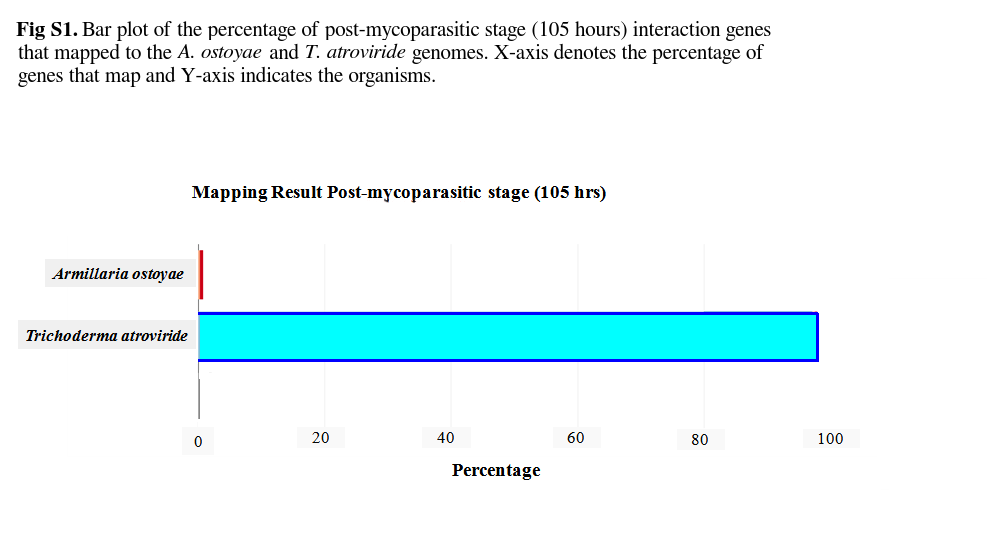

Supplement: Supplemental file 6 — Supplemental material. Download spectrum.04626-22-s0006.tiff, TIFF file, 0.05 MB [file spectrum.04626-22-s0006.tiff]

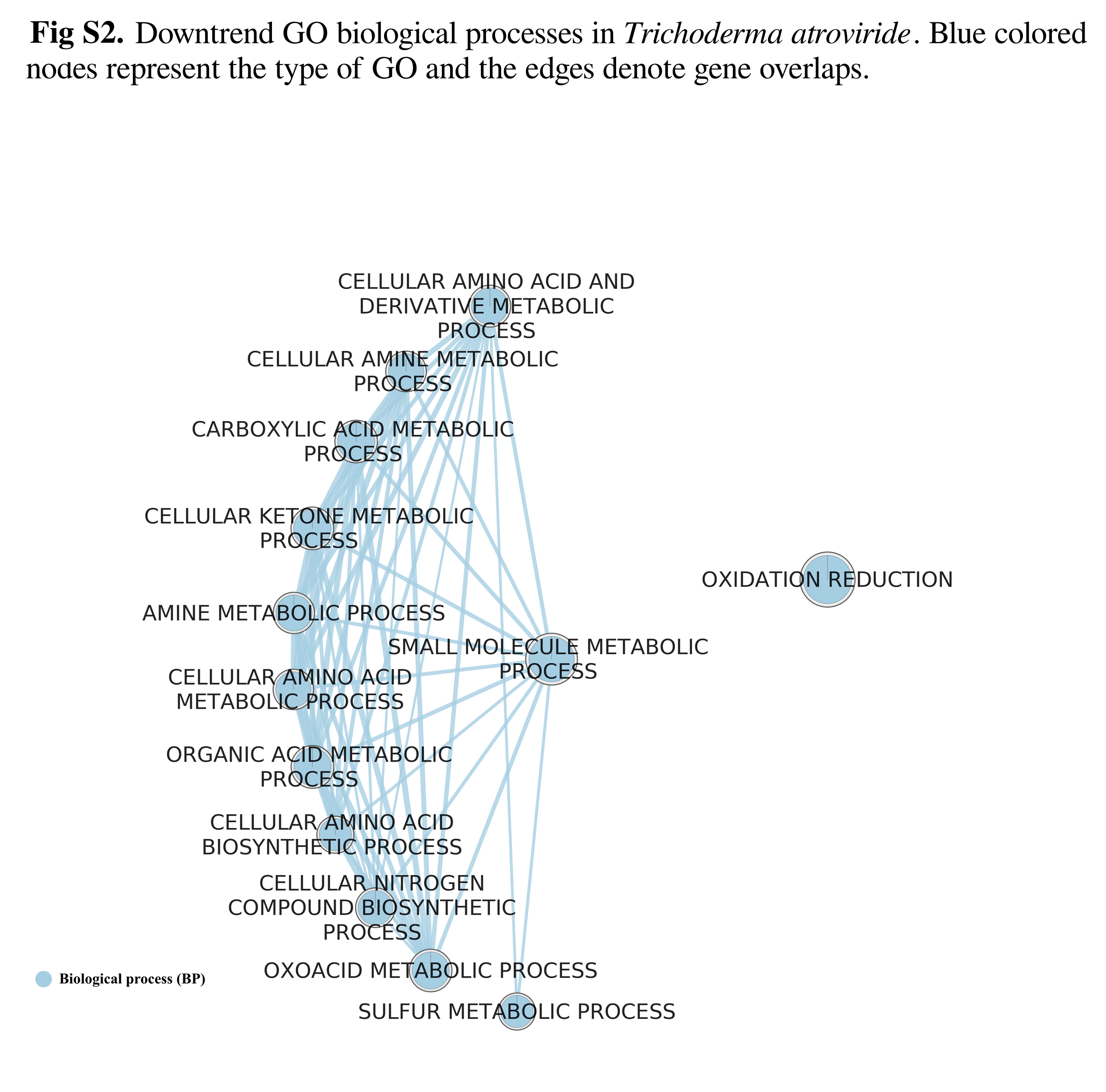

Supplement: Supplemental file 7 — Supplemental material. Download spectrum.04626-22-s0007.tiff, TIFF file, 1.2 MB [file spectrum.04626-22-s0007.tiff]

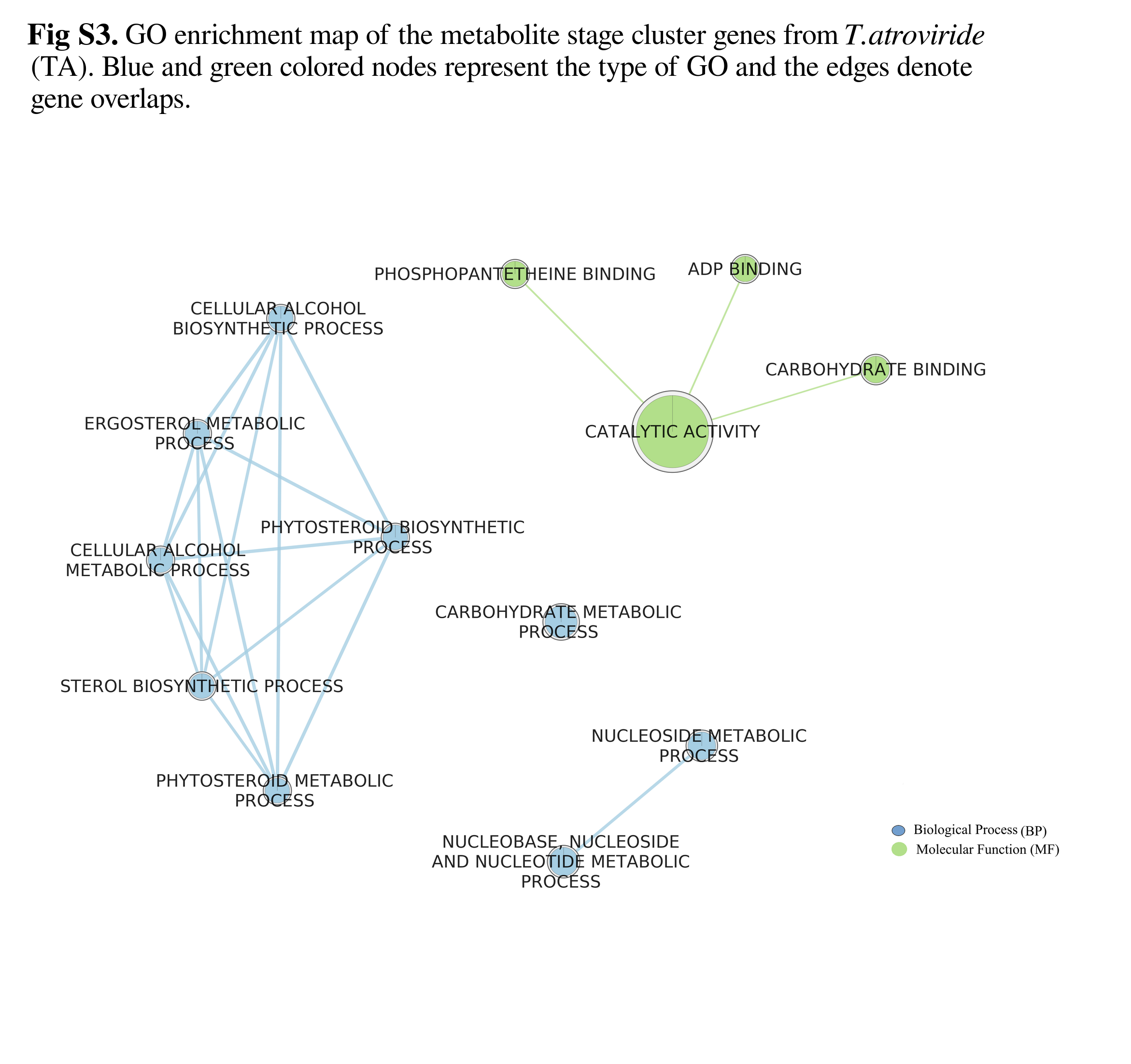

Supplement: Supplemental file 8 — Supplemental material. Download spectrum.04626-22-s0008.tiff, TIFF file, 0.7 MB [file spectrum.04626-22-s0008.tiff]

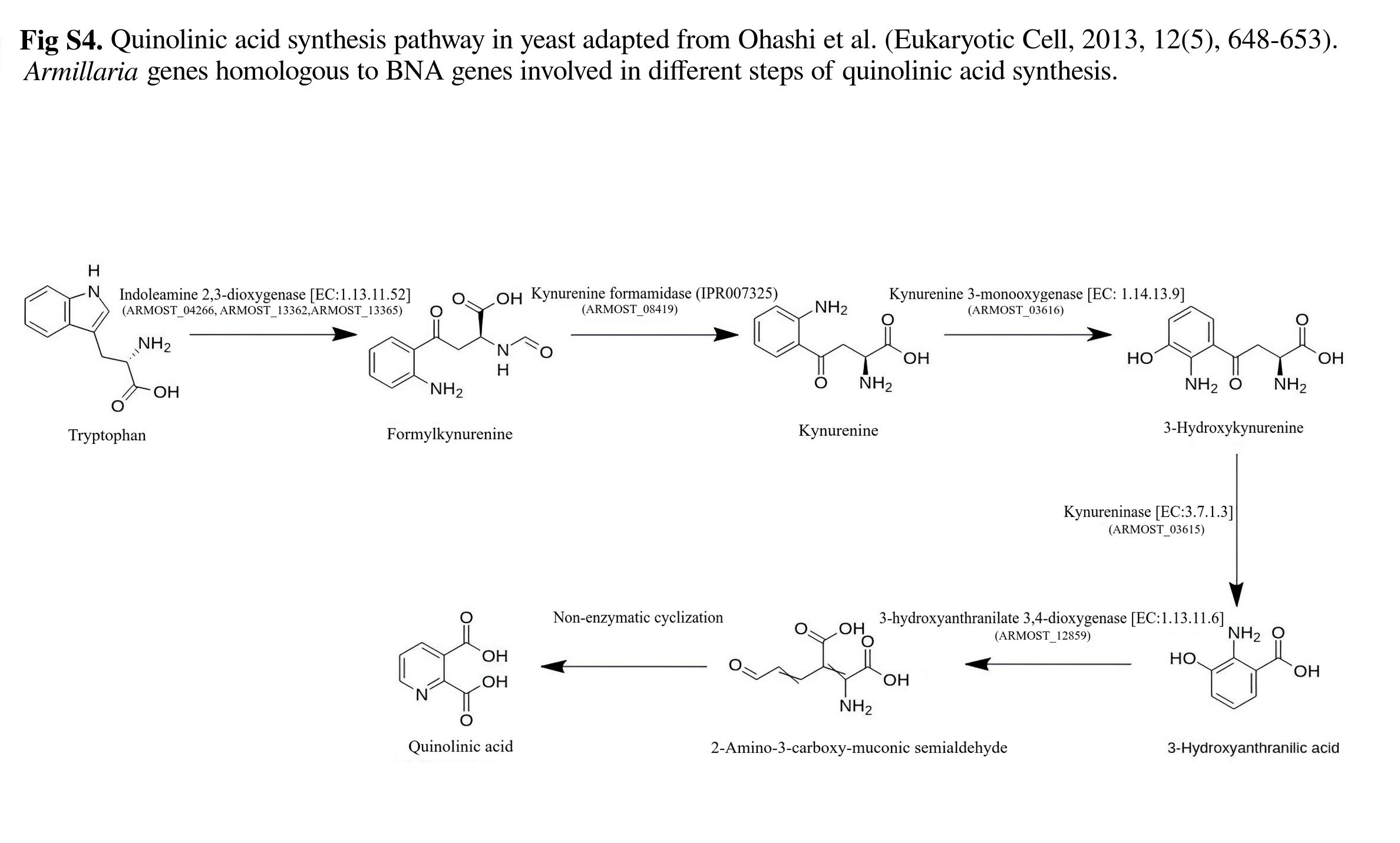

Supplement: Supplemental file 9 — Supplemental material. Download spectrum.04626-22-s0009.tiff, TIFF file, 0.5 MB [file spectrum.04626-22-s0009.tiff]

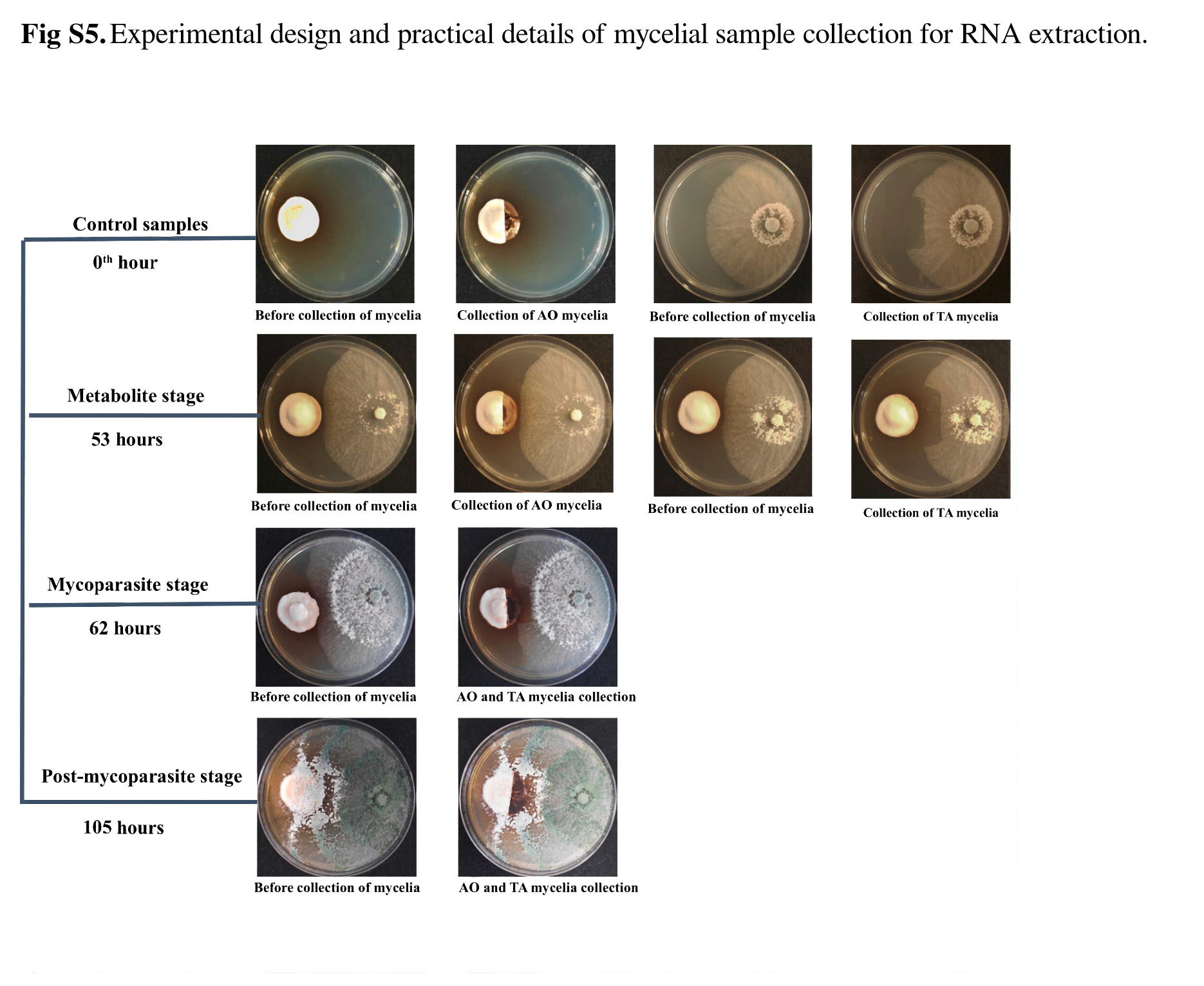

Supplement: Supplemental file 10 — Supplemental material. Download spectrum.04626-22-s0010.tiff, TIFF file, 2.8 MB [file spectrum.04626-22-s0010.tiff]
